# Assessment of serum androgen levels in women with acne vulgaris in Southeastern Nigeria: a cross-sectional study

**DOI:** 10.11604/pamj.2022.41.227.32892

**Published:** 2022-03-18

**Authors:** Chetanna Chioma Anaje, Chinwe Laura Onyekonwu, Gladys Angela Ozoh, Ogochukwu Ifeanyi Ezejiofor

**Affiliations:** 1 Department of Internal Medicine, Nnamdi Azikiwe University Teaching Hospital, Nnewi, Anambra State, Nigeria,; 2 University of Nigeria Teaching Hospital, Ituku Ozalla, Enugu State, Nigeria

**Keywords:** Females, androgen levels, acne vulgaris, androstenedione, testosterone

## Abstract

**Introduction:**

androgens play an important role in the pathogenesis of acne vulgaris. They cause hyperkeratinization of the pilosebaceous follicles and seborrhea. Endocrine diseases characterized by increased levels of androgens often present with acne vulgaris. A correlation between serum androgen levels and acne severity exists, and the assessment of serum androgen levels is therefore essential in women with severe acne vulgaris and treatment resistant acne.

**Methods:**

the study was conducted in the Dermatology Clinic of the University of Nigeria Teaching Hospital, Ituku Ozalla. Seventy females with acne vulgaris and seventy females without acne vulgaris were recruited as subjects and controls respectively. Blood samples were taken from subjects and controls to measure levels of serum testosterone, dehydroepiandrosterone sulfate (DHEAS) and androstenedione. Acne severity was measured using global acne grading system (GAGS).

**Results:**

the median levels of DHEAS and androstenedione (1.20µg/ml and 1.80ng/ml respectively) were higher in subjects than 1.00µg/ml and 1.70ng/ml in controls respectively, although these findings were not statistically significant. There was also no significant difference between the levels of serum testosterone in both the subjects and the controls. No correlation existed between levels of serum androgens and acne severity.

**Conclusion:**

there was no statistically significant difference in the serum androgen levels between the subjects and the control population, and no relationship between androgen levels and severity of acne vulgaris was demonstrated.

## Introduction

Acne vulgaris (AV) is an inflammatory disorder of the pilosebaceous glands of the skin that is characterized by comedones, papules, pustules, nodules and cysts [1]. It is a chronic skin condition that may be complicated by scarring, post-inflammatory hyperpigmentation and keloids. Acne is a common skin disorder that presents frequently both to the internists and the dermatologists. Studies have shown that acne vulgaris may be familial, occurs more frequently in females, although males have more severe disease [2]. It is more common in adolescents and young adults and rare in children less than 10 years and adults older than 50 years [3]. Acne vulgaris is believed to affect more than 80% of young people and accounts for over 14 million visits to physicians per year [4,5]. One study indicated that an estimated 5 to 6 million visits are made to dermatologists every year, resulting in billions of dollars spent on the treatment of acne [6]. The pathogenesis of acne vulgaris involves many factors which include follicular hyperkeratinization, seborrhea, inflammation, and proliferation of *Propionibacterium acnes (P. acnes)* [3]. Serum androgens testosterone, dehydroepiandrosterone sulfate (DHEAS) and androstenedione also play a pivotal role in the development of acne vulgaris [7]. They cause follicular hyperkeratinization and excessive sebum production; consequently, seborrhea stimulates the proliferation of *Propionibacterium acnes* in individuals who have a genetic predisposition to acne [8].

Endocrine diseases such as acromegaly, cushing´s syndrome and adrenal androgen secreting tumors in which increased levels of androgens occur, are characterized by acne vulgaris. Polycystic ovarian syndrome (PCOS) and congenital adrenal hyperplasia (CAH) also present with acne vulgaris [9]. Acne vulgaris may also be a feature of non-endocrine diseases like apert syndrome, synovitis acne pustules hypostasis ostetis (SAPHO) syndrome, behçet syndrome and myogenic arthritis, pyoderma gangrenosum and acne (PAPA) syndrome [10]. Acneiform eruptions may occur as a complication of topical steroid abuse among patients who use steroid-containing skin bleaching products. Elevated serum levels of androgens have been shown to correlate with sebum overproduction and acne [1]. However, acne may occur in subjects with normal levels of serum androgens as a result of hyperresponsiveness of the sebaceous glands to androgens [11]. Studies have looked at the correlation between serum androgens and acne [12-14] but no such study has been done in Southeastern Nigeria. The assessment of serum androgen levels in acne vulgaris patients is essential, and this becomes more important in patients with severe acne vulgaris, treatment-resistant acne vulgaris or late-onset acne vulgaris. Consistently raised levels of serum androgens could be a justification for the use of anti-androgens in the treatment of such categorized patients. The study was undertaken to determine the levels of serum androgens in adult females with acne vulgaris and to correlate the levels of serum androgens with the severity of acne vulgaris among adult females in the dermatology clinic of a tertiary hospital in Enugu State, Nigeria.

**Objective**: this study was conducted to investigate the serum androgen levels in females with acne vulgaris and correlate it with its severity.

## Methods

**Study design:** this study was a prospective, cross-sectional study conducted at the Dermatology Clinic of the University of Nigeria Teaching Hospital (UNTH) in Southeastern Nigeria. It was done between April and October 2016.

**Sample size:** the sample size was calculated using the Kish and Leslie formula [15].


$$n=\frac{Z^{2}pq}{d^{2}}$$

Where n= minimum sample size


$$n=\frac{\left(1.96^{2})(0.043)(0.957\right)}{0.0025}=63.234$$

Z= constant at 95% confidence interval from Z table, p = prevalence of acne vulgaris in Enugu (4.3%) [16], q = 1 - p, d = precision at 95% confidence interval = 0.05. To compensate for a non-response, the formula was used [15].


ns=na

Where n_s_= sample size to be selected, n = original calculated sample size, a = anticipated response = 90%. Calculation:


ns=63.2340.9=70.26

=70 (to the nearest whole number). For the purpose of this study, 140 patients (70 cases and 70 controls) were recruited using a consecutive sampling method. Inclusion criteria for the cases were consenting patients with clinically diagnosed active acne vulgaris (seborrhea and any or all of the other signs of acne such as comedones, papules, pustules, nodules and scarring involving the face, chest and back).

Patients with any of the following criteria were excluded: A) Pregnant women and lactating mothers [13,17]; B) participants who had received oral contraceptive pills (OCPs), anti-androgens, systemic antibiotics and oral retinoids within three months prior to the study [17,18]; C) participants who had received topical antibiotics, benzoyl peroxide, tretinoin, adapalene, tazarotene, topical or systemic corticosteroids within one month prior to study [13,19]; D) participants who had received medications known to affect androgen action or metabolism such as phenytoin, cimetidine, spirinolactone, cyproterone, finasteride [12,20]; E) participants who had received medications that cause acne such as lithium, isoniazid, vitamin B2, B6 or B12, cetuximab. The inclusion criteria of the controls included consenting, aged-matched females without acne vulgaris and not on drugs that affect androgen metabolism or oral contraceptives. Questionnaires were used to obtain the biodata, history of acne and its characteristics, medical and drug history. Participants were examined under proper lighting. The Global Acne Grading System (GAGS) was used to grade the acne severity [21]. It determines the severity of acne by evaluating the type of lesions (comedones, papules, pustules, and nodules) and their anatomic location (forehead, cheeks, nose, chin, chest and back). The scores range from 0 (none) to 1-18 (mild), 19-30 (moderate), 31-38 (severe), and greater than 38 (very severe) [21]. Venous blood samples (five milliters) were collected from the antecubital vein and separated serum was stored at -20°C until assay. There was no requirement for specific timing in the menstrual cycle for drawing the serum sample [22-25]. Serum androgen levels namely testosterone, DHEAS and androstenedione were measured with an enzyme-linked immunosorbent assay (ELISA) manufactured by Demeditec Diagnostics, Germany.

**Ethical consideration:** ethical clearance was sought and obtained from the Ethics Review Board of UNTH, Ituku Ozalla (NHREC/05/01/2008B-FWA 002458-IRB 00002323). Informed written consent was obtained from all participants recruited into the study or their parents/guardian if the patient was between 16 and 18 years of age before enrollment. For the unlettered participants, the informed consent form was read to them in the local language and their thumb prints were obtained in the presence of a witness.

**Statistical analysis:** data analysis was carried out using the Statistical Package for Social Sciences (SPSS™) version 21.0 (SPSS Inc., Chicago, Illinois, USA). The socio-demographic and clinical characteristics of the participants were presented as frequency distribution tables. Mean and standard deviation were computed for normally distributed continuous variable while median and interquartile ranges were computed for skewed continuous variables. Categorical variables were presented as frequencies and percentages. Continuous variables were compared using student´s t-test for normally distributed data while Mann-Whitney U test and Kruskal-Wallis test were performed for skewed data. Association between categorical variables was evaluated using Chi-square and Fisher´s exact test, while the correlation between continuous variables was done using Spearman correlation analysis. A p-value of ≤0.05 was deemed statistically significant.

## Results

The two groups of participants had similar age distributions, the mean ages being 27 ± 7.28 years and 30.4 ± 9.59 years for the females with acne vulgaris and the controls respectively. There was a statistical significance between the educational levels of the subjects and controls. Also, the mean BMI of the subjects and controls was 24.71 kg/m^2^and 24.55 kg/m^2^, and there was no statistically significant difference between the two groups (p = 0.844). On grading the severity of the acne vulgaris with the global acne grading scale, we observed that forty-six subjects (65.7%) had moderate acne while thirteen subjects (18.6%) had mild acne. Eleven subjects (15.7%) had severe acne. We looked at the symptoms of hyperandrogenemia, the commonest symptoms of hyperandrogenemia were hirsutism and alopecia, seen in twenty-five subjects and fourteen subjects (35.7% and 20%) respectively. Table 1 shows the socio-demographic characteristics of the study participants. Table 2 shows the percentage of subjects that had symptoms of hyperandrogenemia. The results of serum androgens are depicted in Table 3. There was no statistical difference between the serum levels of testosterone, androstenedione and DHEAS of the subjects and the controls. There was no significant relationship between serum androgen levels and severity of acne (P > 0.05) (Table 4).

**Table 1:** sociodemographic characteristics of all the study participants

	Subjects	Controls	X^2^	P value
**Age**				
16-19	9 (12.9)	7 (10.0)	10.738	0.097
20-24	23 (32.9)	18 (25.7)		
25-29	9 (12.9)	12 (17.1)		
30-34	19 (27.1)	11 (15.7)		
35- 39	7 (10.0)	9 (12.9)		
40-44	2 (2.9)	4 (5.7)		
45 and above	1 (1.4)	9 (12.9)		
**Educational level**				
University graduate	48 (68.6)	58 (82.9)	11.347	0.010
Secondary school/grade II teachers certificate	8 (11.4)	6 (8.6)		
Primary education	0 (0.0)	3 (4.3)		
No formal education	14 (20.0)	3 (4.3)		
**Occupation**				
Professional/senior civil servant/contractor	11 (15.7)	12 (17.1)	9.218	0.056
Junior civil servants/senior school teacher	1 (1.4)	5 (7.1)		
Junior school teachers/artisan	3 (4.3)	7 (10.0)		
Petty trader/labourer/messenger	2 (2.9)	7 (10.0)		
Unemployed/students	53 (75.7)	39 (55.7)		
**Marital status**				
Single	50 (71.4)	44 (62.9)	2.573	0.462
Married	18 (25.7)	24 (34.3)		
Divorced	1 (1.4)	0 (0.0)		
Widowed	1 (1.4)	2 (2.9)		

**Table 2:** symptoms of hyperandrogenemia

	Frequency	Percent
**Irregular period**		
Yes	7	10.0
No	63	90.0
**Hirsutism**		
Yes	25	35.7
No	45	64.3
**Increase in sex drive (libido)**		
Yes	5	7.1
No	65	92.9
**Alopecia (androgenetic)**		
Yes	14	20.0
No	56	80.0
**Deepening of voice**		
Yes	2	2.9
No	68	97.1

**Table 3:** serum androgen levels between subjects and controls

	Subjects median	Controls median	Mann-whitney u	P value
Testosterone	0.35	0.40	2226.50	0.342
Dheas	1.20	1.00	2021.50	0.073
Androstenedione	1.80	1.70	1993.00	0.057

**Table 4:** correlation of the levels of serum androgens with acne severity

Ordinal variable	Statistics	Testosterone	Dheas	Androste-nedione
Acne vulgaris severit	Spearman's rho coefficient	0.028	0.227	-0.051
	P value	0.819	0.059	0.675
	N	70	70	70

## Discussion

This present study aimed to determine the serum androgen levels of females with acne vulgaris and to correlate it with the severity of acne vulgaris. The age range of the study participants was 16 to 50 years. The subjects aged 20-24 years were about a third and this was followed closely by those aged 30-34. This is similar to findings in previous studies [5,13,17,26] and this could suggest that although acne peaks in the teens, it continues post adolescence. Patients with acne vulgaris and the controls had a mean age of 27.09 ± 7.28 years and 30.40 ± 9.59 years respectively. This could be explained by the fact though serum androgen levels decline before the end of puberty, in some subjects, it persistently remains high. This is further supported by the study of Zaenglein *et al*. [27] where it was observed that acne could persist to the third decade and beyond. Another reason that might account for this relatively high mean age of subjects could be that clients often seek help from other sources including beauticians, drug vendors, chemists and market cream sellers before presenting to the dermatologist. A further reason why patients presented at an older age group as seen in this study is that most parents did not feel that it was worthwhile to take their children with acne vulgaris to the hospital because they felt that acne was just a disease of adolescence which they would outgrow. Thus, they were encouraged to apply over-the-counter or natural products to treat this “normal skin malady”. These patients often presented when they were older and could afford to bring themselves to the hospital while the younger ones were brought by the parent only when the lesions appeared florid. In addition, the use of bleaching creams and comedogenic cosmetics were common within this age group [28]. Although, there are paucity of studies in Nigeria on post-adolescent and adult acne especially in females, the closest was a survey by Akinboro *et al*. [29] in Southwestern part of Nigeria which showed that the mean age of the female participants was 23.48 years which was slightly lower compared to this study.

This may be explained by the fact their study population were recently admitted undergraduates. Other hospital-based studies in Africa reported their mean ages: 23 years in Togo [30] and 25 years in Cameroun [31]. However, these studies were conducted in both males and females. Interestingly, a US study by Collier *et al*. [2] consisting of 1,013 participants of both genders reported a mean age of 48.0 years which was higher than the mean age in this study. A possible explanation may due to their research design which was community based. Moderate acne was the most common grade of acne seen in this study. This agrees with the findings of Rahman *et al*. [13] and Khondker *et al*. [17]. This agrees with the fact that more females suffer moderate acne than severe acne or mild acne. Secondly, females with moderate acne present more to the clinics than females with mild acne. Although Akinboro *et al*. [29] reported that a significant proportion of their study participants had almost clear face. This difference might be attributed to the acne severity scoring scale used in their study (US food and drug administration (US-FDA) 5-category global system of acne classification) which was different from the one used in this study. Okoro *et al*. [32] found that the majority of their participants (88.6%) had mild acne. While their studies were community based, participants were also largely adolescents, it is probable that adolescents generally are less likely to use heavy cosmetics including foundations and oil containing powders. They are also less likely to use skin lightening creams especially while in schools and are less likely to afford the common “mixed creams” sold in the market. Only a few of the subjects (15.7%) had severe acne in this study and this may be explained that hormonal abnormalities, which were not significantly found among the study participants could be implicated. Saka *et al*. [33] reported that almost half (46%) of their acne patients had severe acne. Reasons adduced included the large sample size of their study, the involvement of multiple centers and the inclusion of males with acne vulgaris.

Hirsutism, which usually causes cosmetic concerns and embarrassment for affected females, was the commonest symptom of excessive serum androgens in this study. This is consistent with the findings by Karrer-Voegeli *et al*. [34]. A possible explanation could be that in the locality where this study was carried out, hirsutism was traditionally stigmatising as there was a folklore that hirsute females were usually wicked. Thus, females with hirsutism often seek help for epilation of the unwanted hair. The second most common symptom of hyperandrogenemia observed in the subjects was androgenetic alopecia. Although this finding was at variance with the study by Alan *et al*. [35] alopecia was the third most common presentation of excess androgen levels. Their larger sample could probably account this difference. The implication of hyperandrogenemia in the pathogenesis of acne underscores the importance of assessing serum levels of androgen especially in older women with acne vulgaris [36,37]. Some studies have showed a significant difference between the serum androgen levels of acne patients and the controls [13,35,38]. It was observed that the median levels of serum DHEAS and androstenedione were higher in subjects though the values were within the normal range. However, this study did not reveal any statistically significant differences between the serum androgen levels of the subjects and the controls. This finding is consistent with the study done in the United Kingdom by Darley *et al*. [39]. Furthermore, a study by Aizawa *et al*. [40] found no significant difference in serum testosterone, free testosterone and dihydrotestosterone levels between acne patients and controls. However, they reported the levels of DHEAS was significantly higher in acne patients than in controls. The difference between their study and this study was that their sample size was small (15 participants) who were mainly adolescents. Although some researchers have observed that normoandrogenic patients with acne, as well as, hyperandrogenic patients with acne had increased levels of serum androgen metabolites respectively, it is possible by this study that the presence of acne vulgaris might not dependent on the level of serum androgens. It may therefore be re-affirmed that the severity of acne vulgaris is not necessarily dependent on the level of the serum androgens but on the sensitivity of the sebaceous glands to androgens [41]. However, further comparative local studies are needed to assess the serum androgen levels of women with acne and those without acne.

This study did not demonstrate an overall correlation between serum androgen levels and acne severity. Some researchers [25,35,42] reported a positive correlation between serum androgen levels and severity of acne others did not [39,43]. Schiavone *et al*. [38], in their study of 24 females with acne and 24 controls, reported a significant difference between the serum androgens levels of their subjects and controls, however, no correlation was seen between the severity of acne and the levels of serum androgens. In a study conducted by Levell *et al*. [44], titled “acne is not associated with abnormal plasma androgens” noted that none of the serum androgens except dihydrotestosterone correlated with acne severity. A possible reason for the difference could be that their subjects were aged 12-24 years while in study they were 16 years and above. Although androgens play a role in the pathogenesis of acne, other factors such as gender, genetic factors, family history, diet, the use of comedogenic cosmetics, emotional stress and psychological factors are increasingly important determinants of the severity of acne. Sebaceous gland hypersensitivity to circulating androgens is also believed to be a major factor in the development of acne [36]. The factors discussed above may explain why androgen levels are inconsistent in patients with acne vulgaris irrespective of the severity. Cibula *et al*. [43] found that the severity of acne was not related to serum androgen levels because their patients with severe acne had lower levels of free testosterone, a finding further corroborated by Henze *et al*. [37] who also observed that most of their subjects with severe acne had normal serum androgen levels. In fact, George *et al*. [45], concluded that hormonal therapy was beneficial to both women with hyperandrogenemia and normal androgen levels thus highlighting the importance of hormonal treatment in acne patients irrespective of the serum androgen levels. Although this study did not show a positive correlation between serum androgen levels and severity of acne vulgaris, the importance of serum androgen assay is still appropriate and the awareness should be created especially in cases of treatment resistant acne and acne associated with symptoms of hyperandrogenism such as hirsutism, androgenic alopecia, irregular periods and sudden onset acne.

**Limitations:** this was a clinic-based study. A population study may have more robust outcome and the findings more generalizable. The assay of other serum androgenic parameters like free testosterone, dihydrotestosterone and sex hormone binding globulin (SHBG) might have given a wider androgenic profile.

**Recommendations:** this study has raised questions that would need further research; larger scale studies will further elucidate the role of androgens among women with acne who have normal androgenic values. Further work comparing the degree of response of anti-androgen treatment between women with abnormal androgenic and normal androgenic parameters may give more and better insight the role of androgens among women with acne. Population based research is desirable as it may give a better representation of the serum androgen levels and the effect of acne on the quality of life of women.

## Conclusion

Although this study did not show a difference between serum androgen levels in subjects and controls, it does not undermine the importance of hormonal assay, especially in patients with treatment resistant acne, persistent acne and with other clinical signs of hyperandrogenemia. There was no correlation between the androgen levels with the degree of severity of acne in our subjects. However, the small sample size may make it difficult to generalize this finding. Large-scale studies in this area may shed more light on the role of androgens in the severity of acne vulgaris.

### What is known about this topic

- Serum androgens play an important role in the pathogenesis of acne vulgaris;
- It is essential to assess serum androgen levels in women with severe acne, persistent acne or PCOS.

### What this study adds

- The difference in serum androgen levels were not statistically significant between women with acne vulgaris and women without acne;
- Acne vulgaris may be dependent on the degree of sensitivity of the sebaceous glands to serum androgens and not the levels of serum androgens.
